# A Functional Data Analysis-Based Framework for Modeling and Multi-Objective Optimization of Sustained-Release Drug Delivery Systems

**DOI:** 10.3390/pharmaceutics18060756

**Published:** 2026-06-21

**Authors:** Hao Ren, Mengchen Han, Yuchao Qiao, Yu Cui, Chongqi Hao, Yiming Lou, Gaomin Jing, Qiankun Liu, Lang Yang, Li Zheng, Lixia Qiu

**Affiliations:** 1 Department of Health Statistics, School of Public Health, Shanxi Medical University, Jinzhong 030600, China; 18435147669@163.com (H.R.); h18305693977@163.com (M.H.); haochongqi@163.com (C.H.); 15512382201@163.com (Y.L.); 15035741604@163.com (L.Z.); 2 Shanxi Key Laboratory of Biomedical Data and Statistics, Shanxi Medical University, Jinzhong 030600, China; 3 Key Laboratory of Coal Environmental Pathogenicity and Prevention, Shanxi Medical University, Ministry of Education, Taiyuan 030001, China

**Keywords:** sustained-release formulation, functional data analysis, mixture experimental design, NSGA-III

## Abstract

**Objectives**: An integrated methodological framework was developed for modeling and multiobjective optimization of sustained-release drug delivery systems. **Methods**: The cumulative release percentage was fitted as a function curve, and functional principal component analysis was subsequently used to transform the function curves into functional principal component scores (FPCs). FPCs were then treated as dependent variables, while the proportions of the formulation factors were used as independent variables to construct Scheffé polynomial regression models. Finally, Non-dominated Sorting Genetic Algorithm III (NSGA-III) was applied to perform multi-objective optimization. **Results**: FPC1, FPC2, and FPC3 captured 95.18%, 4.39%, and 0.32% of the total variation, respectively. Corresponding Scheffé polynomial regression models were established, including quadratic models for FPC1 (adjusted *R*^2^ = 0.751, AIC = 168.557) and FPC2 (adjusted *R*^2^ = 0.592, AIC = 119.302), and a special cubic model for FPC3 (adjusted R^2^ = 0.597, AIC = 64.574). The NSGA-III algorithm generated a Pareto optimal set, yielding stable formulation compositions with mean (SD) values of *X*_1_ = 0.123 (0.015), *X*_2_ = 0.821 (0.032), *X*_3_ = 0.012 (0.017), and *X*_4_ = 0.045 (0.015). The corresponding FPCs were −41.787 (2.544), 10.009 (0.168), and 8.264 (0.010) for FPCs1–FPCs3, respectively. The reconstructed cumulative release percentages were 42.471 (1.661), 52.623 (2.868), 69.942 (1.200), 84.275 (1.010), and 93.330 (0.832), demonstrating good agreement with the target release profiles. **Conclusions**: The integrated FDA–Scheffé–NSGA-III framework provides a robust and effective approach for accurately modeling release behavior and optimizing sustained-release formulations.

## 1. Introduction

Sustained-release formulations are drug delivery systems designed to continuously release drugs over an extended period following administration, thereby providing prolonged therapeutic effects [1,2]. Compared to conventional formulations, sustained-release formulations offer several advantages, including the ability to maintain relatively stable drug concentrations at the site of action, reduce peak-to-trough fluctuations in blood drug concentrations, and decrease the dosage and frequency of administration. This, in turn, mitigates adverse effects and enhances patient compliance [3,4]. Consequently, rational experimental design, precise mathematical modeling, and appropriate optimization methods are essential for the development of drug delivery systems in sustained-release formulations.

In sustained-release drug delivery systems, because the formulation mass is fixed, each component is expressed as a proportion and must satisfy the constraint that their sum equals one, known as the mixture constraint [5,6]. This constraint results in a linear relationship among the component variables (independent variables), thereby preventing them from being independent [7]. According to the Pharmacopeia of the People’s Republic of China (ChP), in vitro release testing for sustained-release formulations generally requires that the total cumulative release rate of the drug reach at least 90% [8]. Specifically, at least three representative time points are typically selected from the release curve: the initial phase to assess whether burst release occurs, an intermediate time point to characterize the drug release kinetics, and a terminal time point to evaluate whether drug release is essentially complete. Therefore, when using the cumulative release rates at each time point as quality evaluation indicators (dependent variables), the data exhibited the typical characteristics of repeated measurements. To ensure that the cumulative release rates at different time points simultaneously meet the requirements of the ChP, the problem of optimizing the composition ratios of the sustained-release system essentially constitutes a multi-objective optimization problem [1,9,10].

Owing to the mixture constraints, the most rational design method is the mixture design, which seeks the optimal formulation by manipulating the percentage ratios of the components [11]. Hetal used a D-optimal mixture design to optimize the formulation of a clobetasol propionate-loaded microemulsion-based gel [12]. Peerapattana employed a D-optimal mixture design to optimize metronidazole sustained-release films [13]. Scheffé polynomial regression is widely used for mathematical modeling in mixture design [14].

In sustained-release drug delivery systems, the cumulative release percentage at multiple time points is commonly used as the primary evaluation metric [3]. In traditional modeling approaches, the cumulative release at each time point is often treated as a separate response variable, and individual regression models are constructed. However, this approach has several methodological limitations [15,16]. First, the cumulative release rates at different time points were inherently correlated. Second, modeling and optimizing each time point separately may potentially result in acceptable performance at individual time points, whereas the overall release curve deviates from the desired sustained-release characteristics. Third, this approach fails to fully exploit the continuous nature of the release profile over time, leading to inefficient use of the available data. To address these issues, methods such as generalized estimating equations (GEE) and quadratic inference functions (QIF) have been introduced to account for the correlation structure among time points [17,18]. However, when only a small number of discrete time points is available, these methods have limited capability to fully capture the overall dynamic behavior of drug release in sustained-release formulations.

Essentially, the cumulative release percentage at different time points can be regarded as realizations of a curve or function defined over a continuous time domain. Therefore, treating these observations as discrete measurements at individual time points inherently leads to information loss. Functional data analysis (FDA) provides a natural and systematic statistical framework for addressing this issue. FDA is a collection of statistical techniques for answering questions such as “What are the main ways in which the curves vary from one curve to another?” [19]. Functional Principal Component Analysis (FPCA) plays a fundamental role in the analysis of functional data by providing an efficient and interpretable representation of infinite-dimensional observations. Specifically, FPCA extracts a set of orthogonal principal component functions that capture the dominant patterns of variation in the sample curves, thereby revealing both expected structural features and potentially unexpected patterns [20]. Through this decomposition, functional observations can be approximated by a finite number of principal component values, achieving significant dimensionality reduction while preserving the essential information in the data [20,21]. Furthermore, the proportion of variance explained by the leading components provides a natural measure of the intrinsic complexity of the functional data [21]. Functional principal component scores (FPCs) serve as a bridge between infinite-dimensional functional space and finite-dimensional statistical modeling. They not only achieve effective dimensionality reduction but also preserve the major information contained in the cumulative-release curves.

Multi-objective optimization is another key technique for improving the overall performance of sustained-release drug-delivery systems. The Non-dominated Sorting Genetic Algorithm III (NSGA-III) is an improved multi-objective evolutionary algorithm based on NSGA-II [22]. Building upon the non-dominated sorting framework, the algorithm incorporates reference points, ideal points, and adaptive normalization strategies for solution selection, enabling the population to achieve a more uniform distribution in the objective space while maintaining good convergence performance, thereby further enhancing the global search capability and stability of the algorithm [23].

In summary, a systematic methodological framework is proposed for sustained-release formulation in this study. Within this framework, the release curves were fitted as function curves, and FPCA is subsequently used to transform the continuous functions into FPCs. FPCs were then treated as dependent variables, while the proportions of the formulation factors were used as independent variables to construct Scheffé polynomial regression models. Finally, NSGA-III was applied to perform multi-objective optimization. This framework provides a general mathematical modeling and decision-support platform for the design and process optimization of sustained-release formulations. This enables a methodological transition from traditional pointwise optimization based on discrete time points to the optimization of the entire cumulative release curve, thereby improving the model robustness, predictive accuracy, and overall development efficiency.

## 2. Materials and Methods

### 2.1. Materials

This study conducted statistical modeling and optimization analysis based on experimental data from the literature regarding metronidazole sustained-release films [13]. The original formulation was designed based on a D-optimal mixture design, which is particularly suitable when the experimental region of interest is irregular. The amounts of metronidazole (Metro, *X*_1_), polycaprolactone (PCL, *X*_2_), hydroxypropyl methylcellulose (HPMC, *X*_3_), and glyceryl monostearate (GMS, *X*_4_) were selected as the formulation factors (independent variables). The experimental ranges of the formulation factors are presented in Table S1. The responses of interest (dependent variables) were the cumulative percentage of metronidazole sustained-release films released at days 1, 2, 3, 4, and 5 (*Y*_1_, *Y*_2_, *Y*_3_, *Y*_4_, and *Y*_5_, respectively). There is a total of 20 formulations prepared. The experimental protocol and results for the metronidazole sustained-release films are presented in Table 1.

As shown in Table 1, the sum of the formulation factors is 1, and the cumulative percentage at the third time point in Protocol 14 is lower than that at the second time point, indicating a clear measurement error.

### 2.2. Methods

#### 2.2.1. Functional Data Analysis

FDA provides a methodology for modeling the release process of the metronidazole sustained-release film. FDA models the release process as a function curve, offering a more comprehensive representation of the data compared to traditional discrete point analyses [24,25]. First, B-spline functions are selected as the basis functions, and the number of basis functions is determined using the MSE-elbow method. Due to measurement errors in the experimental data, a smoothing method is applied to fit the cumulative release curve of the metronidazole sustained-release film. To balance the goodness of fit and smoothness, a regularization approach is introduced, where the smoothness of the function curve is measured by the integral of the squared second derivative. The smoothing penalty parameter is selected using the Generalized Cross Validation (GCV) criterion. Since the cumulative release curve of the metronidazole sustained-release membrane is monotonically increasing, monotonic constraints are applied during the modeling process to ensure the release curve remains monotonically increasing. Finally, least squares estimation is used for parameter estimation.

FPCA is one of the main methods in the FDA. FPCA is used to identify the primary modes of variation in the data and determine how many of them are significant [20,21]. FPCA decomposes the observed release curves into orthogonal components, each capturing a specific mode of variation in the release curves. By projecting the functions onto a set of principal components, FPCA enables dimensionality reduction while retaining the key characteristics of the release curves. The FPCs are obtained by projecting each release curve onto the eigenfunctions, which represent the directions of maximum variance. FPCs quantify the contribution of each principal component to the individual release curve, allowing us to represent the original high-dimensional data in a lower-dimensional space.

In this study, the number of principal components is determined based on the cumulative variance contribution, and the principal component scores are calculated as the dependent variable.

#### 2.2.2. Mixture Experiments Modeling

In the design of mixture experiments, there are constraints on the proportion of formulation factors, and the Scheffé polynomial model is used for modeling [6]. The Scheffé model can be expressed in various forms, such as linear models, quadratic models, and special cubic models, to describe different levels of interaction between the formulation factors [14]. In practical modeling, the selection of the appropriate model is based on a comprehensive decision-making process involving statistical tests, model diagnostics, and the specific requirements of the problem at hand. In this study, the fitting performance of different Scheffé models is compared to determine the most suitable model form.

#### 2.2.3. Multi-Objective Optimization

Multi-objective optimization is a critical step in the optimization process of drug delivery systems for sustained-release formulations. The global objective function in multi-objective optimization consists of multiple sub-objective functions, which are mutually constrained and conflicting; the resulting set of solutions is referred to as a Pareto optimal solution.

In mixture experiments, due to the presence of constraints, the constraint problem must first be resolved during the multi-objective optimization process. The Exterior Penalty Function Method is typically employed [26]. This method is a classic approach to handling constrained optimization problems. Its fundamental principle involves introducing a penalty term into the objective function to transform the original constrained optimization problem into an equivalent unconstrained optimization problem [27]. Specifically, by constructing an extended objective function that includes a penalty term, the degree of constraint violation is quantified as a penalty term, which is then optimized alongside the original objective function.

Non-dominated Sorting Genetic Algorithm III (NSGA-III) is an improved multi-objective evolutionary algorithm proposed by Deb et al. in 2014, based on NSGA-II [23]. The core idea is to select individuals using reference points, ideal points, and adaptive normalization techniques on the basis of non-dominated sorting. This approach allows the population to maintain good convergence while achieving a more uniform distribution in the objective space, thereby enhancing the algorithm’s global optimization ability and stability.

#### 2.2.4. Software and Parameter Settings

FDA was performed using the fda package in R. The NSGA-III algorithm was implemented using the Optimization Toolbox in MATLAB R2022a. NSGA-III parameter settings: single-point crossover probability Pc = 0.8, mutation probability Pm = 0.05, initial population size N set to 100, and number of generations set to 200. The algorithm was run independently 15 times for each set of parameters to ensure the reliability and reproducibility of the results.

## 3. Results

### 3.1. Monotone Smoothing

**Figure 1:**
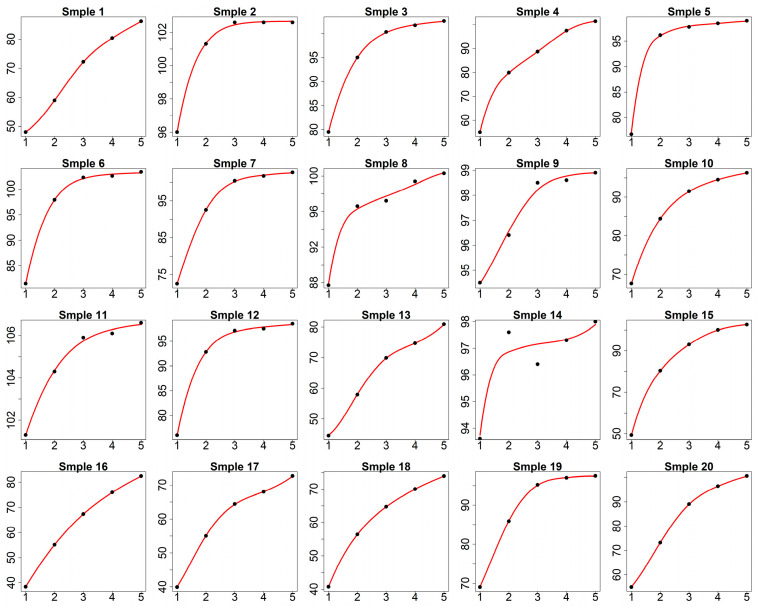
Release curve of a metronidazole sustained-release film. The x-axis represents time, and the y-axis represents the cumulative release percentage of the metronidazole sustained-release film.

The number of B-spline basis functions is seven, and the smoothing parameter is 10^−2^ (Figures S1 and S2). As shown in Figure 1, all metronidazole sustained-release films’ release functions strictly satisfy the monotonicity constraint, with significant variation in the release functions across the different protocols. Among them, Protocols 1, 13, 16, 17, and 18 have release functions that approximate a straight line (indicating a nearly constant release rate), while the other protocols generally show a faster release rate in the early phase, a slower release rate in the middle phase, and a slower release rate in the later phase (the mean function follows a similar pattern). The standard deviation function indicates that the variance of the release functions decreases over time (Figure S3). Table S2 presents the coefficients and fitting errors for the release curves of each protocol. Protocol 14 has the largest fitting error (SSE = 1.147), whereas Protocols 10, 15, 16, and 18 show relatively smaller fitting errors.

### 3.2. Functional Principal Component Analysis

**Figure 2:**
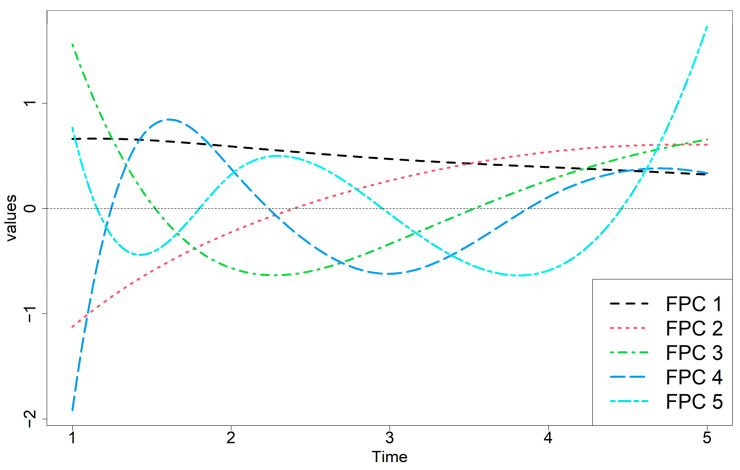
Release curve of a metronidazole sustained-release film. FPC: functional principal component. FPC 1 (black), FPC 2 (red), FPC 3 (green).

Figure 2 displays five FPC (weight function) for the cumulative release percentages of the metronidazole sustained-release films after the mean across all 20 protocols has been removed from each protocol’s cumulative release percentages. Table S3 presents the cumulative variance contribution of the principal components of the release function. FPC1 (weight function) captures 95.18% of the total variation, indicating that this type of variation strongly dominates all other types of variation. Although FPC1 remains positive throughout the entire time, its weight decreases over time. This suggests that the variation in the cumulative release percentages across the protocols diminishes as the release progresses. The protocol for which the FPCs are high will have much greater than average the early phase, combined with a greater later phase, and the two highest FPCs are in fact assigned to Protocols 11 and 2. Unsurprisingly, the largest negative score is assigned to Protocols 17 and 18.

FPC2 accounts for only 4.39% of the total variation and consists of a positive contribution for the middle-later phase and a negative contribution for the early phase, therefore corresponding to a measure of approximately uniformity of cumulative release percentages through time. However, the weight in the early phrase is a little greater than in the later phrase. On this component, the highest FPCs go to Protocols 15 and 20, because they will be much lower than average in the early phase, combined with a greater middle-later phase. Protocols 17 and 18, on the other hand, have greater cumulative release percentages in the early phase and receive large negative second component scores.

FPC3 explains only 0.32% of the total variation, which is a very small proportion and consists of a positive contribution for the early phase, a negative contribution for the middle phase, and a positive contribution for the later phase. The middle phase plays an important role in FPC3. Consequently, the highest FPCs are for Protocol 1, with much-lower-than-average values in the middle phase. The largest negative score is assigned to Protocol 5, with a much greater than average in the middle phase.

FPC4 and FPC5 account for very small proportions of the variation, since they are required to be orthogonal to the first three as well as to each other. The scores of the first three functional principal components are presented in Table 2.

### 3.3. Modeling Results of Mixture Experiment

Based on the results from FPCA, the first three FPCs were selected as dependent variables for building Scheffé polynomial models. In addition to using model evaluation criteria to choose the best model, nested analysis of variance (ANOVA) was also applied.

For FPCs1, a quadratic Scheffé model was selected because the ANOVA showed no significant statistical differences between the quadratic and both the linear and cubic models (linear vs. quadratic, *p* = 0.244, quadratic vs. cubic, *p* = 0.904). However, Table 3 shows that the quadratic model had superior evaluation metrics compared to the linear and cubic models.

For FPCs2, a quadratic Scheffé model was chosen as well. The ANOVA indicated a statistical difference between the linear and quadratic models (*p* = 0.007), but no significant difference between the quadratic and cubic models (*p* = 0.113). As shown in Table 3, although the cubic model having the best evaluation metrics, the quadratic Scheffé model was preferred for its simplicity.

For FPCs3, a cubic Scheffé model was selected. As shown in Table 3, both the linear and quadratic models showed negative adjusted *R*^2^ values, indicating poor fit, while the cubic model produced the best evaluation metrics.

### 3.4. Results of the Multi-Objective Algorithm Optimization

#### 3.4.1. Function Transformation

The Exterior Penalty Function Method is employed to convert an optimization problem with a constant-sum constraint into an unconstrained problem. For the cumulative release percentages of the metronidazole sustained-release film, the target values are 40%, 55%, 70%, 85%, and 100%. These data are transformed into FPCs: −38.233, 10.955, and 8.291. FPCs are then used as the optimization targets, from which the target function is formulated.

(1) $$Q_{1}=abs\left(-38.2329-FPCs_{1}\right)+\rho P\left(x\right)$$

(2) $$Q_{2}=abs\left(10.9554-FPCs_{2}\right)+\rho P\left(x\right)$$

(3) $$Q_{3}=abs\left(8.2908-FPCs_{3}\right)+\rho P\left(x\right)$$

#### 3.4.2. Results of Optimization

The optimization results for each objective function using the NSGA-III algorithm are presented in Table S4. The mean and standard deviation for the formulation factors are as follows: *X*_1_ is 0.123 (0.015), *X*_2_ is 0.821 (0.032), *X*_3_ is 0.012 (0.017), and *X*_4_ is 0.045 (0.015). The mixture component values obtained from the optimization algorithm were substituted into the mathematical model (FPCs_1_, FPCs_2,_ and FPCs_3_) to derive the FPCs. The mean and standard deviation for FPCs are as follows: FPCs1 is −41.787 (2.544), FPCs2 is 10.009 (0.168), FPCs3 is 8.264 (0.010). The results of a one-sample *t*-test indicate that all three FPCs show statistically significant differences from the target function principal component scores (FPC1: −38.233; FPC2: 10.955; FPC3: 8.291). In the literature [13], the Desirability Function method was used to identify Protocol 1 as the optimal solution. The FPCs for this protocol were calculated. The results indicate that the FPCs for Protocol 1 exhibit slight deviations from the target FPCs (Table 4).

In this study, an alternative method was used to compare the optimization results, specifically by utilizing Equation (S18a–e) from the Supplementary Information (which approximates the release curve using FPCs) to calculate the cumulative release percentages for each optimization protocol. Table S5 presents the reconstruction errors. The MSE and RMSE for *Y*_1_ are relatively higher, whereas the errors at the remaining time points are all below one. The mean and standard deviation for the cumulative release percentages are as follows: *Y*_1_ is 42.471 (1.661), *Y*_2_ is 52.623 (2.868), *Y*_3_ is 69.942 (1.200), *Y*_4_ is 84.275 (1.010), and *Y*_5_ is 93.330 (0.832). Compared to the optimal solution in the literature [13], the NSGA-III optimization results are closer to the target cumulative release percentages of the metronidazole sustained-release film (Table S6).

## 4. Discussion

This study presents a methodological framework for sustained-release drug delivery systems that integrates functional data analysis (FDA) into the modeling and optimization of mixture experiments. FDA was used to represent the release curves as function curves rather than discrete observations, providing a more comprehensive description of the release process and enabling a better characterization of its underlying dynamics and temporal trends. To further reduce dimensionality and identify the principal sources of variation in the release profiles, functional principal component analysis (FPCA) was applied. FPCA decomposes the observed release curves into a set of orthogonal components, each capturing a distinct mode of variation. By projecting the functional data onto these principal components, FPCA achieves effective dimensionality reduction while preserving the essential features of the release behavior. In this study, the optimization is conducted within the reduced-dimensional subspace defined by the retained FPCs, which capture the dominant variation in the cumulative release profiles, while the residual component is treated as random noise and does not participate in the optimization procedure [20,21]. This framework not only simplifies the data structure but also provides insight into the main patterns governing variability in the sustained-release process.

The NSGA-III algorithm provides a robust and effective framework for the multi-objective optimization of metronidazole sustained-release film. Although the optimized FPCs differ statistically from the target values, the low variability indicates good stability and reliability. Compared with the Desirability Function approach reported in the literature [13], NSGA-III yields solutions that better balance multiple release characteristics, as reflected by smaller deviations from target FPCs. More importantly, reconstruction of the cumulative release percentage shows that the optimized solutions are consistently closer to the target release percentages across all time points. Using the FPC scores reported in Table 2, the cumulative release percentages at the original five time points were reconstructed. Table S6 shows that the reconstructed profiles closely match the original experimental release data, demonstrating strong consistency between the functional representation and the observed system behavior. For selecting solutions from the Pareto optimal set, one may either choose a single representative solution or consider the average of multiple solutions [28,29,30]. In this study, the first eight solutions are all regarded as preferable candidates.

Compared with the formulation reported in reference [13], the optimal solutions obtained by NSGA-III exhibit an increased proportion of *X*_1_ (metro) and a decreased proportion of *X*_3_ (HPMC). Notably, these adjustments are highly consistent with the known mechanisms governing drug release behavior [31,32]. Metronidazole, as the active pharmaceutical ingredient, directly determines the drug loading within the sustained-release system. An increased drug content generally leads to a higher concentration gradient, which in turn promotes drug diffusion and contributes to an increased cumulative release percentage. In contrast, HPMC is a hydrophilic polymer widely used as a gel-forming matrix in sustained-release formulations [33,34]. Upon contact with aqueous media, HPMC hydrates and forms a viscous gel layer that acts as a barrier to drug diffusion. A reduction in HPMC content weakens the gel structure, thereby decreasing the diffusional resistance and accelerating drug release. Consequently, lowering the proportion of HPMC can enhance the cumulative release of the drug, particularly by mitigating excessive retardation effects.

The FPCA results provide important insights into the underlying variability of the cumulative release behavior of the metronidazole sustained-release films. FPC1, accounting for 95.18% of the total variation, clearly dominates the overall release dynamics, indicating that the primary source of variability across protocols is associated with differences in the overall release level. The consistently positive weight function, coupled with a decreasing trend over time, suggests that inter-protocol differences are most pronounced in the early stage of release and gradually diminish as the system approaches completion. This implies that formulation factors mainly influence the initial release behavior, while later-stage release becomes more uniform across protocols, likely governed by diffusion-controlled mechanisms [35,36]. FPC2, although explaining a much smaller proportion of variance (4.39%), captures the trade-off between early and middle-to-late phase release. This reflects differences in release kinetics rather than total release extent. Protocols with higher FPC2 scores exhibit burst release characteristics in the middle-late phase. In contrast, protocols with negative scores tend to show incomplete release characteristics. Therefore, this component provides a useful descriptor for evaluating release uniformity and temporal distribution, which are critical for maintaining stable therapeutic levels. FPC3, explaining only 0.32% of the variation, represents subtle differences in the middle phase release behavior. Despite its limited contribution, it highlights that variations in the middle stage can still differentiate specific protocols, suggesting that fine-tuning of formulation parameters may influence transitional release dynamics. FPC4 and FPC5 contribute negligibly to the overall variation, indicating that the first three components sufficiently capture the essential features of the release profiles. From a practical perspective, this justifies the use of a reduced number of principal components for subsequent modeling and optimization, thereby simplifying the analysis without significant loss of information. Overall, the FPCA results demonstrate that the variability in drug release is predominantly driven by early-stage behavior and overall release magnitude, while secondary components reflect differences in release kinetics and uniformity. These findings provide a theoretical basis for formulation optimization, particularly in controlling burst release and achieving sustained and stable drug delivery.

The selection of appropriate Scheffé polynomial models for the FPCs reflects a balance between goodness-of-fit, statistical significance, and model parsimony. For FPC1 and FPC2, quadratic models provided an adequate fit without significant improvement when extending to cubic terms, indicating that higher-order interactions contribute limited additional explanatory power. In particular, FPC1, which captures the dominant variation in the release function, is well described by a quadratic model, consistent with moderate nonlinear interactions among formulation factors governing the main release behavior [37]. For FPC2, although the cubic model showed slightly improved evaluation metrics, the improvement was not statistically significant; therefore, the quadratic model was preferred to maintain model simplicity and stability [38]. In contrast, for FPC3, a cubic Scheffé model was selected because both linear and quadratic models yielded negative adjusted R^2^ values, indicating inadequate model fit. Overall, this modeling strategy ensures a reasonable compromise between accuracy and parsimony, thereby enhancing the robustness and interpretability of the mixture modeling framework and providing a reliable basis for subsequent optimization and formulation design.

For the monotonic smoothing of the cumulative release percentage of the metronidazole sustained-release film, we used B-spline basis function (*K* = 7) with a smoothing parameter of 10^−2^ (*λ* = 0.01). Although *K* = 7 is slightly larger than the number of observation points (n = 5), this does not lead to an over-parameterized or ill-posed problem due to the inclusion of a roughness penalty, which ensures that the estimation remains well-posed even when the basis dimension is relatively large relative to the sample size. Within this framework, the effective degrees of freedom are governed not by *K*, but by the smoothing parameter *λ*, which can be characterized through the trace of the hat matrix. The smoothing parameter was selected using generalized cross-validation (GCV) to achieve a balance between data fidelity and smoothness of the estimated trajectory, and it corresponds to the minimum GCV score among a set of candidate values, indicating optimal predictive performance under the adopted criterion. Importantly, in functional data analysis with sparse observations, relatively small values of *λ* are often expected, as excessive smoothing may overshrink functional variability and obscure meaningful temporal patterns. Therefore, *λ* = 0.01 reflects a data-driven trade-off rather than an arbitrary specification.

Overall, a systematic optimization framework for sustained-release drug delivery systems was established by integrating FDA, Scheffé polynomial regression, the Exterior Penalty Function Method, and NSGA-III. This framework provides an effective approach for analyzing and optimizing sustained-release formulations. Although the optimized results demonstrated closer agreement with the target release profiles compared with the reference study, experimental validation was not performed in this study, and we are actively seeking suitable collaborators to address this limitation in future work. Moreover, no external validation using independent datasets was conducted to assess the predictive ability of the regression models, as no additional relevant datasets were available for this study. In addition, the present work primarily focused on enhancing cumulative release percentages, while other critical pharmaceutical attributes, such as tensile strength, drug stability over time, and content uniformity, warrant further investigation. Collectively, these findings highlight the utility of the proposed strategy in balancing multiple formulation objectives and provide a practical and interpretable basis for sustained-release system design.

## 5. Conclusions

In this study, a novel methodological framework integrating FDA, Scheffé polynomial regression, the Exterior Penalty Function Method, and NSGA-III was developed for the design of sustained-release films. The framework ultimately yields a Pareto optimal set, from which one may either select a single representative solution or consider the average of multiple solutions.

## Figures and Tables

**Table 1:** The experimental protocol and results for the metronidazole sustained-release films.

Protocol	Formulation Factors				Measured Response (n = 3)				
	*X*_1_	*X*_2_	*X*_3_	*X*_4_	*Y*_1_	*Y*_2_	*Y*_3_	*Y*_4_	*Y*_5_
1	0.050	0.921	0.000	0.029	48.0 ± 1.3	58.9 ± 2.5	72.2 ± 2.6	80.4 ± 2.0	86.2 ± 3.1
2	0.051	0.649	0.200	0.100	96.0 ± 7.1	101.3 ± 6.8	102.6 ± 7.0	102.6 ± 6.8	102.2 ± 7.1
3	0.200	0.700	0.000	0.100	79.5 ± 3.8	95.0 ± 3.9	100.3 ± 43	101.7 ± 3.7	102.6 ± 4.3
4	0.162	0.744	0.055	0.038	55.0 ± 1.3	79.9 ± 0.5	88.6 ± 0.8	97.4 ± 1.7	101.3 ± 1.7
5	0.200	0.590	0.200	0.010	76.7 ± 0.6	96.2 ± 0.8	97.8 ± 1.5	98.5 ± 1.0	99.0 ± 1.9
6	0.200	0.700	0.000	0.100	81.5 ± 3.3	97.9 ± 4.3	102.3 ± 4.3	102.6 ± 4.4	103.4 ± 3.5
7	0.200	0.623	0.109	0.067	72.5 ± 5.4	92.5 ± 3.2	100.4 ± 4.3	101.7 ± 4.4	102.1 ± 4.8
8	0.136	0.601	0.200	0.063	87.7 ± 1.4	96.6 ± 1.8	97.2 ± 1.6	99.4 ± 1.1	100.3 ± 0.7
9	0.200	0.500	0.200	0.100	94.5 ± 2.2	96.4 ± 2.0	98.5 ± 2.1	98.6 ± 2.2	98.9 ± 1.9
10	0.055	0.808	0.037	0.100	67.6 ± 1.2	84.4 ± 1.6	91.5 ± 2.7	94.5 ± 2.2	96.3 ± 2.1
11	0.200	0.500	0.200	0.100	101.3 ± 4.2	104. 3 ± 4.2	105.9 ± 4.2	106.1 ± 4.4	106.6 ± 3.5
12	0.096	0.715	0.089	0.100	76.0 ± 5.5	92.8 ± 6.2	97.1 ± 5.3	97.5 ± 6.0	98.5 ± 5.4
13	0.050	0.921	0.000	0.029	44.5 ± 0.6	57.9 ± 2.4	69.9 ± 0.4	74.7 ± 2.1	80.9 ± 2.2
14	0.051	0.649	0.200	0.100	93.6 ± 4.5	97.6 ± 5.2	96.4 ± 6.3	97.3 ± 5.0	98.0 ± 6.1
15	0.050	0.761	0.189	0.000	49.3 ± 1.1	80.4 ± 3.0	93.0 ± 6.5	100.0 ± 8.3	102.6 ± 8.0
16	0.077	0.833	0.086	0.004	38.4 ± 0.9	55.1 ± 1.9	67.3 ± 3.4	75.9 ± 4.1	82.4 ± 5.3
17	0.182	0.818	0.000	0.000	39.9 ± 1.9	55.0 ± 3.6	64.4 ± 4.5	68.0 ± 2.7	72.7 ± 3.3
18	0.182	0.818	0.000	0.000	40.7 ± 4.0	56.4 ± 4.1	64.7 ± 3.9	70.0 ± 3.6	73.9 ± 3.5
19	0.144	0.691	0.163	0.032	69.0 ± 3.8	85.9 ± 4.0	95.2 ± 4.0	97.0 ± 4.2	97.5 ± 3.9
20	0.194	0.690	0.115	0.000	54.9 ± 6.3	73.1 ± 8.1	89.0 ± 1.3	96.4 ± 1.6	100.6 ± 1.5

**Table 2:** Functional principal component scores for each protocol.

Protocol	FPCs1	FPCs2	FPCs3
1	−36.504	1.448	3.183
2	30.466	−4.960	0.988
3	20.625	1.707	−0.740
4	−2.403	8.357	1.421
5	18.877	−2.708	−2.156
6	24.652	1.195	−1.375
7	16.999	5.346	−1.958
8	21.371	−4.838	0.942
9	22.545	−6.878	1.623
10	2.395	1.316	−0.761
11	37.304	−3.613	2.157
12	14.623	−0.889	−1.856
13	−42.455	−2.363	−0.050
14	21.596	−8.500	1.169
15	−0.617	13.601	−1.247
16	−46.010	0.842	1.518
17	−51.901	−7.481	−2.176
18	−49.225	−7.005	−1.249
19	6.347	3.583	−1.527
20	−8.684	11.839	2.093

FPCs: functional principal component scores.

**Table 3:** Evaluation metrics for the FPC1, FPC2, and FPC3 models.

FPCs	Model	Adjust *R*^2^	AIC
FPCs1	Linear	0.601	175.359
	Quadratic	0.751	168.557
	Special Cubic	0.642	173.554
FPCs2	Linear	−0.093	136.411
	Quadratic	0.592	119.302
	Special Cubic	0.772	105.484
FPCs3	Linear	−0.144	85.073
	Quadratic	−0.221	88.972
	Special Cubic	0.597	64.574

FPCs: functional principal component scores; AIC: Akaike information criterion.

**Table 4:** NSGA-III optimization results compared with Literature 13.

	Protocol	*X*_1_	*X*_2_	*X*_3_	*X*_4_	FPCs1	FPCs2	FPCs3
NSGA-III	1	0.111	0.852	0.009	0.029	−38.928	10.149	8.262
	2	0.107	0.856	0.009	0.029	−39.099	10.150	8.263
	3	0.112	0.850	0.009	0.029	−39.156	9.523	8.221
	4	0.123	0.839	0.008	0.029	−39.207	9.931	8.266
	5	0.123	0.837	0.009	0.031	−39.245	10.177	8.261
	6	0.123	0.836	0.009	0.031	−39.431	10.177	8.262
	7	0.124	0.832	0.009	0.035	−39.450	10.185	8.261
	8	0.108	0.864	0.008	0.020	−39.692	10.227	8.266
	9	0.114	0.837	0.009	0.041	−40.571	9.886	8.267
	10	0.109	0.842	0.008	0.041	−41.002	9.932	8.267
	11	0.142	0.733	0.083	0.041	−41.780	9.954	8.267
	12	0.145	0.788	0.010	0.057	−41.821	9.960	8.267
	13	0.168	0.768	0.019	0.044	−42.478	9.934	8.267
	14	0.120	0.811	0.003	0.066	−43.683	9.944	8.267
	15	0.120	0.822	0.002	0.055	−44.118	9.955	8.268
	16	0.127	0.804	0.005	0.063	−44.262	9.976	8.268
	17	0.119	0.816	0.004	0.061	−45.076	9.935	8.268
	18	0.109	0.816	0.007	0.068	−45.154	9.962	8.268
	19	0.125	0.800	0.006	0.068	−45.492	10.267	8.267
	20	0.137	0.816	0.006	0.041	−46.085	9.952	8.268
Literature 13	1	0.050	0.869	0.062	0.019	−33.294	8.826	1.453
	2	0.062	0.831	0.097	0.010	−29.61	9.876	0.889
	3	0.086	0.796	0.118	0.000	−30.765	7.402	0.043
	4	0.100	0.771	0.129	0.000	−26.255	6.154	−0.375
	5	0.195	0.737	0.068	0.000	−26.689	4.847	−0.661
	6	0.094	0.753	0.134	0.019	−10.474	6.918	−0.001
	7	0.121	0.707	0.098	0.075	−1.595	16.128	−13.438

## Data Availability

No new data were created or analyzed in this study.

## Supplementary Materials

The following supporting information can be downloaded at https://www.mdpi.com/article/10.3390/pharmaceutics18060756/s1: Table S1: Experimental range for formulation factors; Table S2: Coefficient of the release curve for a metronidazole sustained-release film; Table S3: Principal component of the release function of metronidazole sustained-release film; Table S4: NSGA-III optimization results for target function; Table S5: Reconstruction errors of FPCs of metronidazole sustained-release film; Table S6: NSGA-III optimization results compared with Literature 13; Figure S1: The relationship K between and MSE; Figure S2: The relationship between log(λ) and GCV; Figure S3: Mean and standard deviation functions of the release curve for metronidazole sustained-release films.

## Abbreviations

The following abbreviations are used in this manuscript:

Metro	Metronidazole
PLC	Polycaprolactone
HPMC	Hydroxypropyl Methyl Cellulose
GMS	Glyceryl Monostearate
