# Supplementary material for: A Functional Data Analysis-Based Framework for Modeling and Multi-Objective Optimization of Sustained-Release Drug Delivery Systems
Source: Pharmaceutics. 2026 Jun 21;18(6):756. doi: 10.3390/pharmaceutics18060756 (PMC13307162; doi:10.3390/pharmaceutics18060756)
Supplement: Supplementary file 1 [file pharmaceutics-18-00756-s001.zip › pharmaceutics-4349168-supplementary.pdf]

A Functional Data Analysis-Based Framework for Modeling and Multi-Objective Optimization of Sustained-Release Drug Delivery Systems

S1. Experimental range for formulation factors.

Table S1. Experimental range for formulation factors.

Formulation factors	Experimental range	
	Lower level	Upper level
X_1(%Metro)	5	20
X_2(%PCL)	50	95
X_3(%HPMC)	0	20
X_4(%GMS)	0	10

Metro: metronidazole; PCL: polycaprolactone; HPMC: hydroxypropyl methylcellulose; GMS: glyceryl monostearate

S2. Mixture experiment

A mixture experiment is an important type of experimental design used to investigate the effect of changes in the percentage of each component on a dependent variable. Mixture experiments can be divided into two categories based on whether there are upper and lower limits for the proportions of each component[1,2]. Category 1 has no restrictions,

$$\begin{cases} x_i \geq 0,\ i = 1,2,\cdots,q \\ x_1 + x_2 + \cdots x_q = 1 \end{cases}, \tag{S1}$$

x_i indicates the percentage of each component, q indicates the number of components. Category 2 has restrictions,

$$\begin{cases} L_i \leq x_i \leq U_i,\ i = 1,2,\cdots,q \\ x_1 + x_2 + \cdots x_q = 1 \end{cases}, \tag{S2}$$

L_i indicates lower limit, U_i indicates upper limit. This study belongs to the Category 2. Detailed values are provided in Table S1.

Mixture experiments are subject to the constraint that the component proportions sum to 1, which induces dependence among the components and requires a reparameterization of traditional polynomial regression models. Accordingly, the Scheffé polynomial model is commonly adopted for modeling mixture data. Depending on the polynomial order, Scheffé models can be classified into Linear, Quadratic, and Special Cubic forms, which are used to capture different degrees of interaction among the mixture components[1].

$$\text{Linear: } y = \beta_1 x_1 + \beta_2 x_2 + \cdots \beta_p x_p, \tag{S3}$$

$$\text{Quadratic: } y = \sum_{i=1}^{p} \beta_i x_i + \sum_{1 \leq i < j \leq p} \beta_{ij} x_i x_j \tag{S4}$$

$$\text{Special Cubic: } y = \sum_{i=1}^{p} \beta_i x_i + \sum_{1 \leq i < j \leq p} \beta_{ij} x_i x_j + \sum_{1 \leq i < j < k \leq p} \beta_{ijk} x_i x_j x_k \tag{S5}$$

The coefficients β in the Scheffé polynomial model have clear practical interpretations. The Linear coefficients represent the expected response when component i is present alone,

whereas the Quadratic and Special Cubic coefficients reflect the interactions among components; positive values indicate synergistic effects, while negative values indicate antagonistic effects. In practice, the selection of Linear, Quadratic, and Special Cubic models is not arbitrary, but is determined based on statistical testing, model diagnostics, and practical considerations. In this study, Scheffé models of different orders were compared with respect to their goodness-of-fit to determine the most appropriate model specification.

S3. Functional Data Analysis

S3.1. Functional fitting of discrete data

(1) Basis expansion smoothing

Functional Data Analysis (FDA) aims to represent discrete observations as continuous functional curves. Commonly used approaches include interpolation and smoothing techniques. Interpolation is appropriate when the observations are assumed to be error-free, whereas smoothing methods are preferred in the presence of measurement error to reduce or eliminate noise[3–6].

Let $y_i = \left(y_1, y_2, \cdots y_n\right)$ represent the observed value vector, where the functional curve $Y_i\left(t\right)$ is subject to measurement error ε_i ,such that

$$y_i = Y_i\left(t\right) + \varepsilon_i, \tag{S6}$$

Consider a set of basis functions $\Phi\left(t\right) = \left(\Phi_1\left(t\right), \cdots, \Phi_k\left(t\right)\right)$,and use a linear combination of these basis functions to approximate the curve $Y_i\left(t\right)$, i.e.,

$$Y_i\left(t\right) = \sum_{k=1}^{K} c_{ik} \Phi_k\left(t\right), \tag{S7}$$

where c_{ik} are the coefficients corresponding to the k-th basis function in the expansion of $Y_i\left(t\right)$.

The goal is to estimate the system parameters c by minimizing the residual sum of squared errors:

$$SMSSE\left(y|c\right) = \sum_{i=1}^{N}\left[y_i - Y_i\left(t\right)\right]^2 = \sum_{i=1}^{N}\left[y_i - \sum_{k=1}^{K} c_{ik}\Phi_k\left(t\right)\right]^2, \tag{S8}$$

This expression can be written in matrix form as:

$$SMSSE\left(y|c\right) = \left(y - \Phi c\right)^T \left(y - \Phi c\right), \tag{S9}$$

where the coefficients c are estimated as:

$$c = \left(\Phi^T \Phi\right)^{-1} \Phi^T y. \tag{S10}$$

Commonly used basis function types include Fourier basis: composed of sine and cosine functions, especially suitable for describing data with periodic patterns; B-spline basis: composed of piecewise polynomials, which can adapt well to the local variations of non-periodic data; and wavelet basis: suitable for capturing local features in the data, such as abrupt changes or high-frequency fluctuations. The infinite-dimensional function-type data is approximated as a sum of a finite number of terms (with K terms), where a larger K results in a higher degree of fit but lower smoothness, and a smaller K leads to a poorer fit but a smoother curve.

(2) Roughness penalty method

When fitting a function curve to discrete observations, in order to balance the smoothness of the function curve and the data fitting accuracy, the concept of regularization is introduced. A commonly used approach is the least squares error (SSE), which represents the degree of fit. To enforce smoothness on the function curve, the second derivative is commonly used as a penalty to control the roughness of the fitted curve. The smoothness penalty is defined as:

$$PEN_2(Y) = \int \left\{ D^2 Y(t) \right\} dt, \tag{S11a}$$

where $D^m(\bullet)$ represents the m-th derivative of the function. A smaller $PEN_2(Y)$ indicates that the curve is closer to a straight line.

The penalized sum of squared error (PENSSE) combines both the fitting degree and the smoothness penalty, defined as:

$$PENSSE_\lambda(Y|y) = \sum \left[y_i - Y_i(t) \right]^2 + \lambda \times PEN_2(Y), \tag{S11b}$$

where λ is the smoothing parameter. The smoothing parameter λ controls the trade-off between fitting and smoothness. A larger λ indicates that the function is more heavily penalized for roughness, while a smaller λ allows the function to fit the data more closely, potentially resulting in overfitting. The optimal choice of λ is typically determined by generalized cross-validation (GCV), which selects the value of λ that minimizes the following criterion:

$$GCV(\lambda) = \left[\frac{n}{n - df(\lambda)} \right] \left[\frac{SSE}{n - df(\lambda)} \right], \tag{S11c}$$

where $df(\lambda)$ represents the degrees of freedom at λ ,and $SSE = \sum [y_i - \hat{y}_i]^2$ is the residual sum of squares.

(3) Monotone constrained functions

In practical applications, individual observations may experience brief declines due to measurement errors or random fluctuations. For example, in Sample 14 of Table S1, the third observation is lower than the second, resulting in a negative growth rate (which corresponds to the second derivative of the function being less than 0), a situation that lacks real-world significance. The smoothing method described above is an unconstrained function approximation approach, making it challenging to naturally enforce shape constraints such as monotonicity and non-negativity during the estimation process.

To ensure monotonicity, this study introduces a smoothing function in the fitting stage to guarantee that the estimated curve maintains monotonic growth. Specifically, let the function $Y_i(t) = \int_{t_0}^{t} \exp[w(u)]\, du$,and the curve is expressed as:

$$y_i = \beta_0 + \beta_1 \int_{t_0}^{t_i} \exp[w(u)]\, du + \varepsilon_i = \beta_0 + \beta_1 \int_{t_0}^{t_i} \exp[\phi(u)' c]\, du + \varepsilon_i, \tag{S12}$$

Where β_0 is the value of the approximating function at t_0 ,For monotonically increasing functions, β_1 could be absorbed into $w(u)$ .However, to accommodate monotonically decreasing functions, we keep β_1 separate and normalize $w(u)$ for numerical stability.

In this study, the cumulative release curve of metronidazole sustained-release membrane is treated as a function curve, which is fitted using B-spline basis functions. To achieve a better

fit for the cumulative release, the study employs the MSE-elbow method to determine the optimal number of basis functions.

S3.2. Functional principal analysis

Functional Principal Component Analysis (FPCA) is similar to multivariate principal component analysis[3–6]. First, define the average function of the function curves

$$\bar{Y}(t) = N^{-1}\sum_i Y_i(t), \tag{S13}$$

and the covariance function at different time points s and t as:

$$v(s,t) = \operatorname{cov}(Y_i(s), Y_i(t)) = (N-1)^{-1}\sum_i \left[Y_i(s) - \bar{Y}(s)\right]\left[Y_i(t) - \bar{Y}(t)\right], \tag{S14}$$

The eigenvalue and eigenfunction are obtained by solving the eigenfunction equation:

$$\int v(s,t)\alpha_i(t)\,dt = \lambda_i \alpha_i(s), \tag{S15}$$

with the conditions that: $\int \alpha_i(t)^2\,dt = 1$ and $\int \alpha_i(t)\alpha_j(t)\,dt = 0$ (orthogonality). $\alpha_i(t)$ is eigenfunction or weight function. λ_i is eigenvalue. The functional principal components scores (FPCS) are obtained by solving the integral equation:

$$\xi_i = \int \alpha_i(t) Y_i(t)\,dt. \tag{S16}$$

The Karhunen-Loève expansion of $Y_i(t)$ is

$$Y_i(t) = \mu(t) + \sum_{l=1}^{\infty} \xi_{il}\alpha_l(t), \tag{S17}$$

where the FPCS $\xi_{il} = \int \alpha_i(t)\left[Y_i(t) - \mu(t)\right]dt$ are independent random variables, $\xi_{il} \sim N(0, \lambda_l)$ .Assuming that the first L eigenfunctions were sufficient to approximate the longitudinal process $Y_i(t)$, a truncated form was given by

$$Y_i(t) \approx \mu(t) + \sum_{l=1}^{L} \xi_{il}\alpha_l(t). \tag{S18a}$$

Equation 18 can be used to approximate the curve $Y_i(t)$.

The determination of the number of FPC is typically based on the scree plot, cumulative variance contribution, or the GCV criterion, balancing between information retention and model simplicity.

S4. Multi-Objective Optimization

(1) Exterior Penalty Function Method

The Exterior Penalty Function Method (EPFM) is a widely used approach for handling constraints in optimization problems. This method transforms a constrained optimization problem into an unconstrained problem by incorporating penalty terms that penalize the violation of constraints[7,8].

In the context of multi-objective optimization, the objective function can be expressed as:

$$Q(x,\rho) = f(x) + \rho P(g(x)). \tag{S18b}$$

where $P(\bullet)$ is the penalty function, and $\rho > 0$ is the penalty parameter. As ρ increases, the solutions that violate the constraints will incur a heavier penalty, thus ensuring that the optimization process gradually approaches feasible solutions.

(2) Non-dominated Sorting Genetic Algorithm III

The basic steps of the NSGA-III algorithm are as follows[9]:

(1).Population Initialization: Based on the characteristics of the optimization problem, parameters such as the number of reference points, maximum number of iterations, crossover probability, and mutation probability are set. The initial parent population P_t is randomly generated, with a size of N .

(2). Population Update with Non-dominated Sorting: Through crossover and mutation operations, the parent population P_t generates offspring population Q_t ,which has the same size N as the parent population. The parent and offspring populations are then combined to form a population of size $2N$,denoted as R_t .A fast non-dominated sorting based on Pareto dominance is performed on R_t ,and the individuals in each non-dominated layer are selected into the next generation population S_t ,ensuring that the best individuals are retained until $S_t \geq N$,thus maintaining the diversity of the population.

(3). Construction of Reference Points and Ideal Points: In the objective space, the reference points are generated by the following formula:

$$H = \left(\frac{M+H-1}{H} \right). \tag{S18c}$$

This formula generates a set of reference points uniformly distributed on a unit hyperplane, enhancing the selection of individuals. At the same time, the minimum values of each objective function in the population S_t are calculated to determine the ideal points:

$$Z_i^{\min} = (Z_1^{\min}, Z_2^{\min}, \cdots Z_M^{\min}). \tag{S18d}$$

(4).aptive Normalization Processing: To eliminate the influence of different objective scales and ranges, the Achievement Scalarizing Function (ASF) is introduced to determine the ideal point in each objective direction, and calculate the linear scalar distance on each axis of the objective space a_i .Its calculation formula is as follows:

$$A_{SF}(x, w) = \max_{x=1,\cdots,m} \frac{f_i'(x)}{w}, x \in S_t, \tag{S18e}$$

$$f_i^n(x) = \frac{f_i'(x)}{a_i - z_i^{\min}},$$

which realizes the transformation of the objective function values.

(5).Relationship Between Individuals and Reference Points: In the post-normalized objective space, each reference point is associated with the original points, forming the corresponding reference lines. During the calculation, each individual in the population S_t is mapped to the perpendicular distance to the different reference lines, and the individual is linked to the closest reference point, providing data for the selection of the next evolutionary boundary.

(6). Calculation of the Smallest Boundary of Reference Points: The smallest boundary is calculated directly in S_t ,where individuals that meet the conditions are selected as the next generation, and the cycle continues until the termination criteria are met.

S5. Result

S5.1. The number of basis functions

To determine the number of basis functions, the smoothing parameter $\lambda = 10^{-8}$ and $K = 5, \cdots, 10$ were used, with the mean squared error (MSE) as the evaluation index. From the Figure S1, it can be seen that as K increases from 5 to 7, the average MSE significantly decreases. However, when $K \geq 7$, the MSE enters a plateau, and further increases in the number of basis functions have limited improvement on the fitting accuracy. Therefore, the number of basis functions was set to $K = 7$.

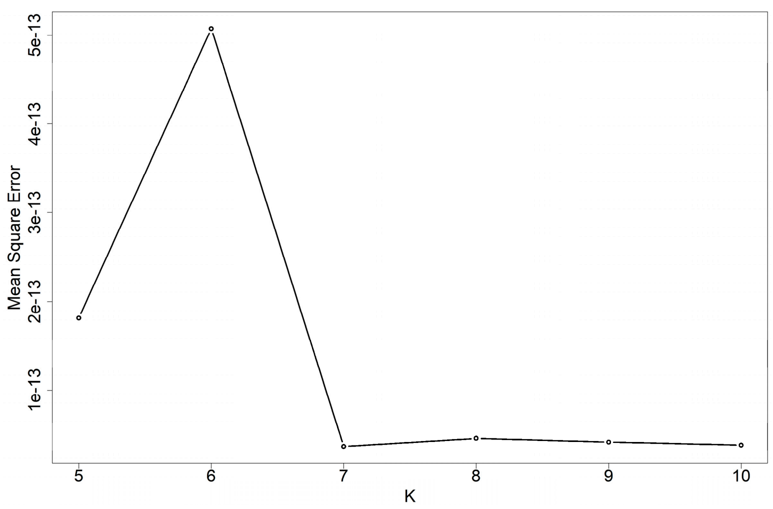

Figure S1. The relationship between K and MSE.

S5.2. Smoothing parameter

The smoothing parameter is selected based on the GCV criterion. As shown in Figure S2, with the increase of $\log(\lambda)$, GCV increases rapidly, indicating that the relase curve becomes increasingly smoother, leading to over-smoothing. Since the data only contains five time points and uses monotone constrained, smaller values of λ would result in unstable estimates, causing near singularities. Therefore, the smoothing parameter was set to $\lambda = 10^{-2}$.

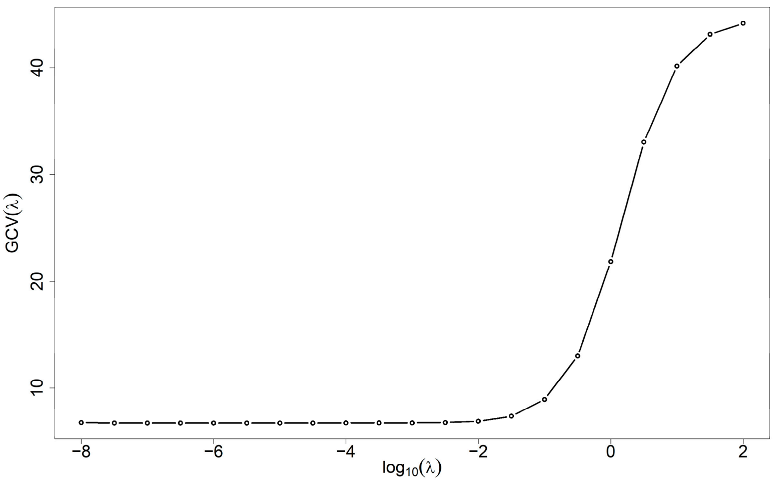

Figure S2. The relationship between $\log(\lambda)$ and GCV.

S5.3. Monotone smoothing

Table S2 presents the coefficients and fitting errors of the release curves for each protocol. Protocol 14 had the largest fitting error (SSE = 1.147), while protocol 10, 15, 16, and 18 had smaller fitting errors.

Table S2. Coefficient of the release curve for a metronidazole sustained-release film.

Formulation	$\beta_{0,i}$	bspl4.1	bspl4.2	bspl4.3	bspl4.4	bspl4.5	bspl4.6	bspl4.7	SSE	MSE
1	47.991	48.072	50.271	58.548	73.465	80.726	84.340	86.190	0.010	0.002
2	95.996	96.012	99.298	101.861	102.553	102.654	102.663	102.666	0.029	0.006
3	79.498	79.518	87.461	96.638	100.674	101.916	102.377	102.537	0.028	0.006
4	55.006	55.154	71.868	80.783	88.251	98.339	100.880	101.308	0.053	0.011
5	76.705	77.156	93.263	96.689	98.168	98.420	98.876	98.911	0.257	0.052
6	81.498	81.525	90.621	99.755	102.453	103.011	103.162	103.206	0.207	0.041
7	72.495	72.561	80.881	95.152	101.073	101.942	102.501	102.555	0.114	0.023
8	87.718	87.900	94.786	96.527	97.717	99.014	100.105	100.349	0.533	0.107
9	94.454	94.468	95.046	96.684	98.452	98.806	98.891	98.894	0.138	0.028
10	67.599	67.614	75.717	85.949	92.040	94.695	95.823	96.281	0.003	0.001
11	101.292	101.296	102.686	104.636	105.874	106.326	106.472	106.521	0.069	0.014
12	75.998	76.030	85.685	94.641	97.157	97.891	98.199	98.351	0.201	0.040
13	44.487	44.706	46.981	59.011	71.420	74.317	77.935	80.815	0.085	0.017
14	93.639	93.737	96.530	96.915	97.190	97.271	97.588	97.893	1.147	0.229
15	49.302	49.308	66.701	82.340	93.740	100.837	102.113	102.643	0.008	0.002
16	38.400	38.408	44.740	55.889	67.918	76.223	80.474	82.398	0.000	0.000
17	39.893	40.006	44.560	56.705	65.324	67.812	70.538	72.642	0.041	0.008
18	40.701	40.701	47.975	57.504	65.118	70.208	72.773	73.898	0.000	0.000
19	68.993	69.103	74.614	87.892	96.453	97.055	97.534	97.407	0.040	0.008
20	54.955	55.091	59.724	74.070	90.817	96.660	99.493	100.556	0.028	0.006

SSE：sum of squared errors；MSE：Mean Squared Error.

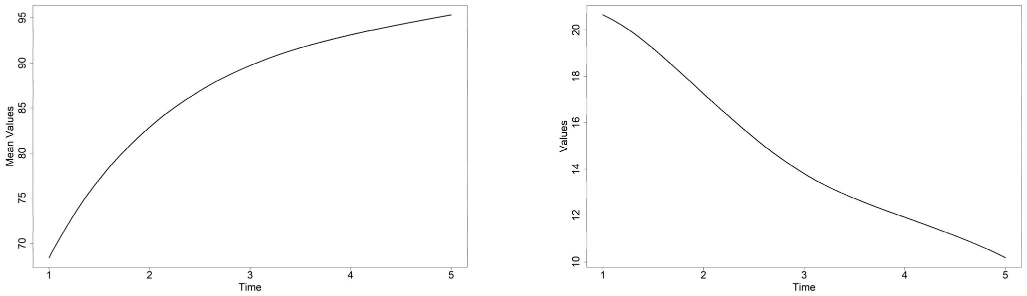

Figure S3. Mean and standard deviation functions of the release curve for metronidazole sustained-release films.

S5.4. Functional Principal Component Analysis

Table S3. Principal component of the release function of metronidazole sustained-release film.

	FPC1	FPC2	FPC3	FPC4	FPC5
eigenvalue	806.982	37.231	2.724	0.847	0.027
FVE(%)	0.95182	0.04391	0.00321	0.00099	0.00003
CFEV(%)	0.95182	0.99573	0.99894	0.99994	0.99998

FPC: Functional principal component; FVE: Fraction of variance explained; CFVE: Cumulative fraction of variance explained.

S5.5. Mixture Experiments Modeling

FPCs1 model is expressed as follows:

$$FPCs_1 = 233.1704 \cdot x_1 - 29.788 \cdot x_2 + 946.219 \cdot x_3 - 8058.976x_4 - 285.149x_1x_2 \\ - 3944.999x_1x_3 + 6485.054x_1x_4 - 844.344x_2x_3 + 9681.742x_2x_4 + 6645.105x_3x_4$$

FPCs2 model is expressed as follows:

$$FPCs_2 = 830.054 \cdot x_1 + 108.306 \cdot x_2 - 593.289 \cdot x_3 - 1340.862x_4 - 117.379x_1x_2 \\ - 474.953x_1x_3 + 1627.530x_1x_4 + 833.864x_2x_3 + 1450.873x_2x_4 + 561.335x_3x_4$$

FPCs3 model is expressed as follows:

$$FPCs_3 = 243.431 \cdot x_1 + 4.504 \cdot x_2 - 199.024 \cdot x_3 + 393.531x_4 - 333.933x_1x_2 \\ - 596.864x_1x_3 - 8268.993x_1x_4 + 224.686x_2x_3 - 505.101x_2x_4 + 7211.977x_3x_4 \\ + 969.176x_1x_2x_3 + 11085.605x_1x_2x_4 + 2766.461x_1x_3x_4 - 10865.259x_2x_3x_4$$

S5.6. Results of the Multi-Objective Algorithm Optimization

Table S4. NSGA-III optimization results for target function.

Protocol	Mixture Component				Target Function		
	X_1	X_2	X_3	X_4	Q_1	Q_2	Q_3
1	0.111	0.852	0.009	0.029	0.695	0.450	0.028
2	0.107	0.856	0.009	0.029	0.867	0.450	0.028
3	0.112	0.850	0.009	0.029	0.923	1.077	0.070
4	0.123	0.839	0.008	0.029	0.974	0.669	0.025
5	0.123	0.837	0.009	0.031	1.012	0.423	0.029
6	0.123	0.836	0.009	0.031	1.198	0.422	0.029
7	0.124	0.832	0.009	0.035	1.217	0.414	0.030
8	0.108	0.864	0.008	0.020	1.459	0.373	0.025
9	0.114	0.837	0.009	0.041	2.338	0.713	0.024
10	0.109	0.842	0.008	0.041	2.769	0.667	0.024
11	0.142	0.733	0.083	0.041	3.547	0.645	0.024
12	0.145	0.788	0.010	0.057	3.588	0.640	0.024
13	0.168	0.768	0.019	0.044	4.245	0.665	0.024
14	0.120	0.811	0.003	0.066	5.450	0.655	0.024
15	0.120	0.822	0.002	0.055	5.885	0.645	0.023
16	0.127	0.804	0.005	0.063	6.029	0.623	0.023
17	0.119	0.816	0.004	0.061	6.843	0.665	0.023

18	0.109	0.816	0.007	0.068	6.921	0.638	0.023
19	0.125	0.800	0.006	0.068	7.259	0.332	0.023
20	0.137	0.816	0.006	0.041	7.852	0.647	0.023

Table S5. Reconstruction errors of FPCs of metronidazole sustained-release film.

	Y_1	Y_2	Y_3	Y_4	Y_5
MSE	3.325	0.220	0.476	0.059	0.204
RMSE	1.823	0.470	0.690	0.243	0.451

Table S6. NSGA-III optimization results compared with Literature 13.

	**Protocol**	X_1	X_2	X_3	X_4	Y_1	Y_2	Y_3	Y_4	Y_5
NSGA-III	1	0.111	0.852	0.009	0.029	44.2	55.97	71.32	85.47	94.33
	2	0.107	0.856	0.009	0.029	44.08	55.87	71.24	85.4	94.28
	3	0.112	0.850	0.009	0.029	44.69	55.01	71.06	85.03	93.85
	4	0.123	0.839	0.008	0.029	44.26	55.86	71.13	85.25	94.11
	5	0.123	0.837	0.009	0.031	43.96	55.78	71.18	85.36	94.25
	6	0.123	0.836	0.009	0.031	43.83	55.67	71.09	85.29	94.19
	7	0.124	0.832	0.009	0.035	43.81	55.66	71.09	85.29	94.19
	8	0.108	0.864	0.008	0.020	43.61	55.51	70.98	85.21	94.14
	9	0.114	0.837	0.009	0.041	43.41	55.06	70.48	84.69	93.65
	10	0.109	0.842	0.008	0.041	43.08	51.8	70.29	84.54	93.54
	11	0.142	0.733	0.083	0.041	42.54	51.34	69.93	84.25	93.3
	12	0.145	0.788	0.010	0.057	42.51	51.31	69.91	84.24	93.29
	13	0.168	0.768	0.019	0.044	42.1	50.93	69.6	83.96	93.06
	14	0.120	0.811	0.003	0.066	41.3	50.22	69.03	83.5	92.68
	15	0.120	0.822	0.002	0.055	41.00	49.96	68.83	83.33	92.55
	16	0.127	0.804	0.005	0.063	40.88	49.87	68.77	83.29	92.52
	17	0.119	0.816	0.004	0.061	40.39	49.4	68.38	82.95	92.23
	18	0.109	0.816	0.007	0.068	40.31	49.35	68.35	82.93	92.22
	19	0.125	0.800	0.006	0.068	39.74	49.08	68.27	82.96	92.3
	20	0.137	0.816	0.006	0.041	39.71	48.8	67.91	82.56	91.92
Literature 13	1	0.050	0.869	0.062	0.019	40.00	60.08	76.39	85.00	90.79
	2	0.062	0.831	0.097	0.010	40.00	62.50	78.38	86.93	92.23
	3	0.086	0.796	0.118	0.000	40.00	63.07	77.18	85.00	89.87
	4	0.100	0.771	0.129	0.000	43.30	66.36	78.95	86.03	90.34
	5	0.195	0.737	0.068	0.000	45.20	66.23	78.95	85.00	88.97
	6	0.094	0.753	0.134	0.019	53.00	75.32	86.31	92.90	95.90
	7	0.121	0.707	0.098	0.075	70.38	87.38	94.20	97.70	99.51

References

1. Myers, R.H.; Montgomery, D.C.; Anderson-Cook, C.M. *Response Surface Methodology: Process and Product Optimization Using Designed Experiments*; John Wiley & Sons, 2016; ISBN 1-118-91603-4.
2. Allan; W; Dickinson Experiments With Mixtures: Designs, Models, and the Analysis of Mixture Data. *Technometrics* **1982**, *24*, 161–162.
3. Jane-Ling Wang; Jeng-Min Chiou; Hans-Georg Müller Functional Data Analysis. *Annual*

Review Statistics and Its Application **2016**, *3*, 257–295, doi:10.1146/annurev-statistics-041715-033624.

4. Ramsay, J.; Hooker, G.; Graves, S. *Functional Data Analysis with R and MATLAB*; Springer New York: New York, NY, 2009; ISBN 978-0-387-98184-0.
5. Ramsay, J.O.; Silverman, B.W. *Functional Data Analysis*; Springer series in statistics; 2. ed., [Nachdr.].; Springer: New York, NY, 2006; ISBN 978-0-387-40080-8.
6. Functional Data Analysis - Welcome! Available online: https://www.psych.mcgill.ca/misc/fda/index.html (accessed on 28 March 2026).
7. Farag, M.H. Application of the Exterior Penalty Method for Solving Constrained Optimal Control Problems. *Expert Review of Hematology* **2013**, *6*, 429–439.
8. Yeniay, Ö. Penalty Function Methods for Constrained Optimization with Genetic Algorithms. *MCA* **2005**, *10*, 45–56, doi:10.3390/mca10010045.
9. Deb, K.; Jain, H. An Evolutionary Many-Objective Optimization Algorithm Using Reference-Point-Based Nondominated Sorting Approach, Part I: Solving Problems With Box Constraints. *IEEE Transactions on Evolutionary Computation* **2014**, *18*, 577–601.
